# Sociodemographic variables associated with risk for diabetic retinopathy

**DOI:** 10.1186/s40842-022-00144-z

**Published:** 2022-10-24

**Authors:** Chan Tran N. Nguyen, Matheos Yosef, Shokoufeh Khalatbari, Anjali R. Shah

**Affiliations:** 1 Michigan State University College of Human Medicine, East Lansing, MI USA; 2 University of Michigan Institute for Clinical and Health Research, Ann Arbor, MI USA; 3 Department of Ophthalmology and Visual Sciences, University of Michigan, Ann Arbor, MI USA; 4 University of Michigan, Kellogg Eye Center, 1000 Wall Street, 48105 Ann Arbor, MI USA

**Keywords:** Diabetic retinopathy, Risk factors, Health disparities, Health outcomes

## Abstract

**Background:**

Several systemic and sociodemographic factors have been associated with the development and progression of diabetic retinopathy (DR). However, there is limited investigation of the potential role sociodemographic factors may play in augmenting systemic risk factors of DR. We hypothesize that age, sex, race, ethnicity, income, and insurance payor have an impact on hemoglobin A1c (HbA1c), body mass index, and systolic blood pressure, and therefore an upstream effect on the development of DR and vision-threatening forms of DR (VTDR).

**Methods:**

Multivariable analysis of longitudinal electronic health record data at a large academic retina clinic was performed. Sociodemographic factors included race, ethnicity, income, and insurance payor. Systemic risk factors for DR included hemoglobin A1c (HbA1c), systolic blood pressure (SBP), and body mass index (BMI). VTDR was identified from encounter diagnostic codes indicating proliferative retinopathy or diabetic macular edema. Patient-reported primary address zip codes were used to approximate income level, stratified into quartiles.

**Results:**

From 2016 to 2018, 3,470 patients with diabetes totaled 11,437 visits were identified. Black patients had higher HbA1c and SBP compared to White patients. White patients had higher BMI and SBP compared to patients of unknown/other race and greater odds of VTDR than the latter. Patients of Hispanic ethnicity had significantly higher SBP than non-Hispanic patients. Low-income patients had higher BMI and SBP than high-income patients and greater odds of VTDR than the latter. Medicaid recipients had greater odds of VTDR than those with Blue Care Network (BCN) and Blue Cross Blue Shield (BCBS) insurance. Medicaid and Medicare recipients had higher SBP compared to BCBS recipients. Finally, both higher HbA1c and SBP had greater odds of VTDR. There were no differences in odds of VTDR between White and Black patients or between Hispanic and non-Hispanic patients.

**Conclusion:**

Significant associations exist between certain sociodemographic factors and well-known risk factors for DR. Income and payor were associated with increased severity of systemic risk factors and presence of VTDR. These results warrant further investigation of how risk factor optimization and disease prevention may be further improved by targeted intervention of these modifiable sociodemographic factors.

**Supplementary information:**

The online version contains supplementary material available at 10.1186/s40842-022-00144-z.

## Background

Diabetic retinopathy (DR) is the leading cause of acquired blindness in the working-age adult population in the United States and is one of the most common causes of preventable blindness globally [1]. Vision changes due to DR present late in disease progression, and 90% of blindness occurrence may be prevented with routine examination [2]. Visual impairment secondary to diseases such as DR remains a major public and global health concern with a significant impact on patient quality of life and workforce productivity.

Several studies have reported an association between social determinants of health and presence of DR, as well as diabetic macular edema (DME) and proliferative DR, both vision-threatening types of DR. A recent publication using a large data registry showed that Black and Hispanic patients had higher proportions of proliferative DR than White or non-Hispanic patients. These authors also reported that sociodemographic factors such as race, ethnicity, and insurance payor were all associated with differences in visual acuity prior to initiating treatment for vision-threatening DR. Furthermore, they found that Black patients were more likely to have more severe DR compared to White patients. The same held true for Hispanic or Latino patients compared to non-Hispanic patients, as well as Medicaid recipients compared to those with private insurance [3].

Physiologic factors such as elevated body mass index (BMI), higher hemoglobin A1c (HbA1c), and higher blood pressure are well documented risk factors for DR, with sustained elevations of these variables associated with increased disease progression [4–10]. There is some literature suggesting that disparities in rates of DR among different cohorts of patients can be attributed to systemic risk factors regardless of social determinants of health such as race and ethnicity. A study by Wong and colleagues showed that although Black and Hispanic patients have increased prevalence of DR and DME compared to White and Chinese patients, differences between these groups decreased when the authors accounted for duration of diabetes and serum fasting glucose [11]. This finding was corroborated in another study showing that increased prevalence and severity of DR in Black patients compared to White patients could be traced to higher severity of systemic risk factors of DR in the former population [12].

However, other research disputes the exclusive contribution of systemic risk factors to severity of progression of DR. The Salisbury Eye Study found that African American patients were 4 times more likely than White patients to suffer visual impairment from DR, raising the question of disparities in prevention and intervention among the two cohorts [13]. Another study concluded that the odds of Black patients developing DR was 2.96 times higher than White patients, even after adjusting for HbA1c, blood pressure, and diabetes treatment [14]. These findings suggest that not only may there be an independent association between sociodemographic factors and rates of DR, but that certain social determinants of health may also contribute to severity of risk factors for DR development and progression.

The ultimate consequence of such a relationship is an upstream compounding effect of sociodemographic factors on the severity and progression of DR, significantly diminishing visual outcomes in certain populations. Thus, a better understanding of how sociodemographic factors are associated with systemic risk factors for DR is vital to disease prevention and earlier, more targeted disease intervention to minimize severity and vision loss. In this single-center retrospective study, we hypothesize that sociodemographic factors such as income, race, ethnicity, and payor have a direct impact on established systemic risk factors for diabetes, such as poor glycemic control, hypertension, and elevated BMI. We believe these ultimately have a compounding upstream effect on development of both DR and vision-threatening forms of DR (VTDR).

## Methods

### Study design

This retrospective review was conducted using data from the Comprehensive Diabetic Retinopathy Program (CDRP) at Kellogg Eye Center. This program was established in 2016 by faculty from Michigan Medicine Ophthalmology and Visual Sciences Retina Clinic in collaboration with faculty from the Metabolism, Endocrinology and Diabetes division with the goal of identifying risk profiles of patients with diabetes and optimizing intervention strategies to reduce adverse outcomes. The program catalogues a comprehensive collection of ocular, non-ocular, and chronic health data. The collection and analysis of this data was approved by the University of Michigan Institutional Review Board (HUM00129794).

Data was collected on all patients with a diagnosis of diabetes presenting to the Kellogg Eye Center retina clinic between July 2016 and June 2018. A total of 3,470 patients with 11,437 visits to the Kellogg Eye Center retina clinic between July 2016 and June 2018 were included. Diagnosis of diabetes was determined by presence of any type of diabetes in the past medical history or electronic health record problem list.

Race and ethnicity data were self-reported. Patients with no race or ethnic identification in the electronic medical record or those whose race could not be determined were included in analysis and considered “unknown”. Race was categorized as Black, White, and Unknown/Other/Mixed. This third cohort included patients identifying as Asian or Pacific islander, Native American, and Mixed race. Ethnicity was categorized as Hispanic, non-Hispanic, and Unknown/refused to identify. Zip codes from the patient’s primary address, along with 2017 United States census data, were used to determine median household income values. Zip codes with median incomes no greater than the first quartile were designated as low income, those no less than the third quartile as high income, and those between these quartiles as medium income. Additional information such as age, sex, and payor (primary insurance coverage provider) were all obtained from the electronic health record.

International Classification of Disease (ICD) codes from patient visits were used to determine whether the patient had vision-threatening forms of DR (VT). Vision threatening disease included the presence of either diabetic macular edema, proliferative diabetic retinopathy, or both. Having DR without these specific diagnoses was considered not vision threatening.

### Statistical analysis

Patient sociodemographic characteristics were summarized as counts and percentages for categorical data and mean ± standard deviation for continuous data. The characteristics considered were baseline age, patient sex, race, ethnicity, median household income category based on zip code, and payor. Both cross-sectional and longitudinal analyses of the risk factors HbA1c, BMI, and SBP were conducted against all the characteristics. The cross-sectional models were performed at first visit, while the longitudinal analyses were carried out using linear mixed models with age at visit or days from first visit as time. Fit diagnostics were performed, and the residual plots did not show any pattern. HbA1c was analyzed using a cross-sectional model as values were only available for 44% of patients. All other risk factors were analyzed longitudinally. All analyses were performed using SAS (version 9.4, SAS Institute, Cary, NC, USA).

## Results

Of the 3470 patients included in the analysis, 41% of patients presented to the clinic only once during the study period. Table 1 demonstrates baseline characteristics of the cohort. The mean age for patients was 62.2 years and 46.5% were female. Racial distribution for the cohort was 73.7% White, 15.3% Black, and 11.0% unknown/other/mixed race. Most patients were non-Hispanic at 90.2%. The income distribution consisted of 26.9% low-income, 48.6% medium-income, and 24.4% high-income patients. The most common insurance payors were Medicare (37.0%) and Blue Cross Blue Shield (23.9%).

**Table 1:** Baseline characteristics

Characteristic	n = 3470
Baseline age, years	62.2 ± 14.6
**Sex**	
Female	1615 (46.5)
Male	1855 (53.5)
**Race**	
Black	530 (15.3)
Unknown/other/mixed	379 (11.0)
White	2548 (73.7)
**Ethnicity**	
Non-Hispanic	3116 (90.2)
Unknown/refused	222 (6.4)
Hispanic	115 (3.3)
**Income**	
Low (≤ Q1)	935 (26.9)
Medium (Q1, Q3)	1687 (48.6)
High (≥ Q3)	848 (24.4)
**Payor**	
BCBS	830 (23.9)
BCN	588 (16.9)
Medicaid	52 (1.5)
Medicare	1,285 (37.0)
Other commercial	715 (20.6)

Values expressed as mean ± SD or n (%). BCBS: Blue Cross Blue Shield; BCN: Blue Care Network

Table 2 shows that in a global cross-sectional multivariable analysis, lower HbA1c was significantly associated with higher age (Beta estimate − 0.02 [-0.03, -0.02], p < 0.001). Higher HbA1c was significantly found in Black patients (Beta estimate 0.40 [0.17, 0.64], p < 0.001). There were no significant associations between HbA1c and sex, income, ethnicity, or insurance payor.

**Table 2:** Baseline cross-sectional models of hemoglobin A1c (HbA1c)

	Univariable (n = 3470)			Multivariable (n = 1541)	
**Effect**	**n Used**	**Estimate [95% CI]**	**p-value**	**Estimate [95% CI]**	**p-value**
Female sex	1548	-0.05 [-0.22, 0.13]	0.60	-0.04 [-0.22, 0.13]	0.62
Age at visit	1548	-0.02 [-0.03, -0.02]	*< 0.001*	-0.02 [-0.03, -0.02]	*< 0.001*
**Income**	1548		*0.02*		0.44
Low (≤ Q1) vs High (≥ Q3)		0.35 [0.11, 0.60]	*0.01*	0.16 [-0.09, 0.41]	0.20
Medium (Q1, Q3) vs High (≥ Q3)		0.17[-0.04, 0.38]	0.11	0.08 [-0.13, 0.29]	0.44
**Race**	1542		*< 0.001*		*< 0.01*
Black vs White		0.45 [0.21, 0.68]	*< 0.001*	0.40 [0.17, 0.64]	*< 0.001*
Unknown/other/mixed vs White		-0.01 [-0.28, 0.25]	0.93	-0.09 [-0.36, 0.19]	0.54
**Ethnicity**	1542		0.13		0.47
Non-Hispanic vs Hispanic		-0.36 [-0.85, 0.13]	0.15	-0.31 [-0.82, 0.20]	0.24
Unknown/refused vs Hispanic		-0.62 [-1.22, -0.01]	*0.045*	-0.36 [-0.98, 0.25]	0.25
**Payor**	1548		*< 0.01*		0.58
BCBS vs Other commercial		-0.35 [-0.63, -0.08]	*0.01*	-0.23 [-0.50, 0.04]	0.10
BCN vs Other commercial		-0.14 [-0.42, 0.14]	0.32	-0.15 [-0.43, 0.12]	0.28
Medicaid vs Other commercial		-0.05 [-0.77, 0.68]	0.90	-0.15 [-0.87, 0.57]	0.68
Medicare vs Other commercial		-0.48 [-0.73, -0.23]	*< 0.001*	-0.17 [-0.43, 0.09]	0.21

BCBS: Blue Cross Blue Shield; BCN: Blue Care Network

Table 3 demonstrates the relationship between sociodemographic factors and BMI. Significant associations with higher BMI were found in females (Beta estimate 1.60 [1.01, 2.19], p < 0.001), unknown/other/mixed race (Beta estimate − 3.54 [-4.54, -2.54], p < 0.001), low-income patients (Beta estimate 1.54 [0.70, 2.39], p < 0.001), and those with Blue Care Network insurance (Beta estimate 0.31 [0.04, 0.59], p = 0.03). Lower BMI was found to be associated with older age (Beta estimate − 0.04 [-0.07, -0.02], p < 0.001). No significant association was found between BMI and ethnicity.

**Table 3:** Longitudinal models of body mass index (BMI)

	Univariable (#Obs = 11,435) (#Subjs = 3470)				Multivariable (#Obs = 9133) (#Subjs = 3452)	
**Effect**	**n Used**	**#Subjs**	**Estimate [95% CI]**	**p-value**	**Estimate [95% CI]**	**p-value**
Age at visit	9134	3470	-0.05 [-0.07, -0.02]	*< 0.001*	-0.04 [-0.07, -0.02]	*< 0.001*
Female sex	9134	3470	1.64 [1.05, 2.24]	*< 0.001*	1.60 [1.01, 2.19]	*< 0.001*
**Income**	9134	3470		*< 0.001*		*< 0.01*
Low (≤ Q1) vs High (≥ Q3)			1.75 [0.92, 2.56]	*< 0.001*	1.54 [0.70, 2.39]	*< 0.001*
Medium (Q1, Q3) vs High (≥ Q3)			0.81 [0.09, 1.53]	*0.03*	0.65 [-0.06, 1.36]	0.07
**Race**	9133	3457		*< 0.001*		*< 0.001*
Black vs White			0.15 [-0.67, 0.96]	0.73	-0.32 [-1.15, 0.50]	0.45
Unknown/other/mixed vs White			-3.27 [-4.24, -2.31]	*< 0.001*	-3.54 [-4.54, -2.54]	*< 0.001*
**Ethnicity**	9133	3453		0.55		0.08
Non-Hispanic vs Hispanic			-0.14 [-1.89, 1.60]	0.87	-1.59 [-3.39, 0.21]	0.08
Unknown/refused vs Hispanic			0.54 [-1.55, 2.63]	0.61	-0.64 [-2.75, 1.46]	0.55
**Payor**	9134	3470		*0.02*		*0.01*
BCBS vs Other commercial			-0.02 [-0.30, 0.25]	0.86	-0.03 [-0.30, 0.25]	0.86
BCN vs Other commercial			0.30 [0.02, 0.57]	*0.04*	0.31 [0.04, 0.59]	*0.03*
Medicaid vs Other commercial			-0.30 [-0.69, 0.09]	0.14	-0.29 [-0.69, 0.10]	0.14
Medicare vs Other commercial			-0.10 [-0.35, 0.15]	0.43	-0.12 [-0.36, 0.13]	0.35

BCBS: Blue Cross Blue Shield; BCN: Blue Care Network

Multivariable analysis of the association between sociodemographic factors and SBP is shown in Table 4. Higher SBP are noted in older patients (Beta estimate 0.25 [0.20, 0.30], p < 0.001), low-income patients (Beta estimate 2.56 [0.60, 4.52], p = 0.01), and Black patients (Beta estimate 6.35 [4.42, 8.28], p < 0.001). Patients with Medicare and Medicaid were also noted to have higher SBP compared to those with Blue Cross Blue Shield (p < 0.01 and p = 0.04, respectively). Lower SBP is associated with non-Hispanic ethnicity (Beta estimate − 7.86 [-11.81, -3.92], p < 0.001) and patients who were of unknown/other/mixed race (Beta estimate − 3.31 [-5.63, -0.99], p < 0.01). There were no significant associations with sex.

**Table 4:** Longitudinal analysis of systolic blood pressure (SBP)

	Univariable (#Obs = 11,437) (#Subjs = 3470)				Multivariable (#Obs = 10,351) (#Subjs = 3452)	
**Effect**	**n Used**	**#Subjs**	**Estimate [95% CI]**	**p-value**	**Estimate [95% CI]**	**p-value**
Age at visit	10,363	3470	0.25 [0.20, 0.29]	*< 0.001*	0.25 [0.20, 0.30]	*< 0.001*
Female sex	10,363	3470	0.04 [-1.35, 1.42]	0.96	-0.51 [-1.88, 0.87]	0.47
**Income**	10,363	3470		*< 0.001*		*0.02*
Low (≤ Q1) vs High (≥ Q3)			4.56 [2.63, 6.49]	*< 0.001*	2.56 [0.60, 4.52]	*0.01*
Medium (Q1, Q3) vs High (≥ Q3)			1.16 [-0.53, 2.85]	0.18	0.43 [-1.25, 2.11]	0.62
**Race**	10,352	3457		*< 0.001*		*< 0.001*
Black vs Whie			6.85 [4.96, 8.75]	*< 0.001*	6.35 [4.42, 8.28]	*< 0.001*
Unknown/other/mixed vs White			-1.72 [-3.94, 0.52]	0.13	-3.31 [-5.63, -0.99]	*< 0.01*
**Ethnicity**	10,352	3453		*< 0.001*		*< 0.001*
Non-Hispanic vs Hispanic			-6.08 [-9.86, -2.30]	*< 0.01*	-7.86 [-11.81, -3.92]	*< 0.001*
Unknown/refused vs Hispanic			-2.89 [-7.55, 1.78]	0.23	-4.01 [-8.75, 0.72]	0.10
**Payor**	10,363	3470		*< 0.01*		*0.02*
BCBS vs Other commercial			-1.36 [-3.10, 0.37]	0.12	-1.14 [-2.87, 0.58]	0.19
BCN vs Other commercial			0.04 [-1.75, 1.82]	0.97	0.15 [-1.63, 1.92]	0.87
Medicaid vs Other commercial			2.94 [-0.55, 6.43]	0.10	2.68 [-0.81, 6.16]	0.13
Medicare vs Other commercial			1.29 [-0.33, 2.91]	0.12	1.28 [-0.33, 2.89]	0.12

BCBS: Blue Cross Blue Shield; BCN: Blue Care Network

Table 5 shows the association between multiple systemic and sociodemographic variables and the presence of VTDR as determined by diagnosis codes. In the multivariable analysis, the odds of having VT- DR were significantly increased for low-income patients (OR 1.57 [1.09, 2.26], p = 0.02), those with higher HbA1c (OR 1.17 [1.09, 1.25], p < 0.001), and those with higher SBP (OR 1.01 [1.01, 1.02], p < 0.001). Those on Medicaid also had higher odds of having VTDR than those on Blue Care Network (p = 0.01) and Blue Cross Blue Shield (p = 0.03). While there was a significantly decreased risk of VTDR in those of unknown/other/mixed race compared to White (OR 0.46 [0.30, 0.70], p < 0.001), there were no statistically significant differences in the odds of having VTDR between White and Black patients (OR 1.09 [0.78, 1.52], p = 0.62) or between Hispanic and non-Hispanic patients (OR 0.62 [0.31, 1.27], p = 0.19).

**Table 5:** Longitudinal analysis of vision-threatening diabetic retinopathy (VTDR)

	Univariable (#Obs = 11,437) (#Subjs = 3470)				Multivariable (#Obs = 4484) (#Subjs = 1704)	
**Effect**	**n Used**	**#Subjs**	**OR [95% CI]**	**p-value**	**OR [95% CI]**	**p-value**
Days from first visit					1.00 [1.00, 1.00]	*< 0.001*
Baseline age	11,433	3470	0.96 [0.95, 0.96]	*< 0.001*	0.96 [0.96, 0.97]	*< 0.001*
Female sex	11,433	3470	0.89 [0.73, 1.07]	0.21	0.98 [0.76, 1.25]	0.84
A1c (LOCF)	5085	1973	1.26 [1.18, 1.34]	*< 0.001*	1.17 [1.09, 1.25]	*< 0.001*
BMI	9132	2675	1.01 [0.99, 1.02]	0.41	0.10 [0.98, 1.01]	0.82
SBP	10,359	3029	1.01 [1.01, 1.01]	*< 0.001*	1.01 [1.01, 1.02]	*< 0.001*
**Income**	11,433	3470		*0.02*		0.05
Low (≤ Q1) vs High (≥ Q3)			1.45 [1.11, 1.89]	*< 0.01*	1.57 [1.09, 2.26]	*0.02*
Medium (Q1, Q3) vs High (≥ Q3)			1.23 [0.97, 1.55]	0.08	1.29 [0.96, 1.73]	0.09
**Race**	11,417	3457		*< 0.001*		*< 0.001*
Black vs White			1.61 [1.24, 2.09]	*< 0.001*	1.09 [0.78, 1.52]	0.63
Unknown/other/mixed vs White			0.65 [0.48, 0.90]	*< 0.01*	0.46 [0.30, 0.70]	*< 0.001*
**Ethnicity**	11,409	3453		*< 0.01*		0.16
Non-Hispanic vs Hispanic			0.65 [0.39, 1.08]	0.10	0.62 [0.31, 1.27]	0.19
Unknown/refused vs Hispanic			0.37 [0.20, 0.70]	*< 0.01*	0.43 [0.18, 1.03]	0.06
**Payor**	11,433	3470		*< 0.001*		*0.04*
BCBS vs Other commercial			0.71 [0.55, 0.92]	*0.01*	0.80 [0.55, 1.17]	0.25
BCN vs Other commercial			0.89 [0.67, 1.18]	0.41	0.69 [0.47, 1.01]	0.05
Medicaid vs Other commercial			3.03 [1.55, 5.92]	*0.001*	2.55 [0.93, 6.99]	0.07
Medicare vs Other commercial			0.80 [0.63, 1.01]	0.06	0.97 [0.68, 1.39]	0.86

BCBS: Blue Cross Blue Shield; BCN: Blue Care Network; BMI: body mass index; LOCF: last observation carried forward; SBP: systolic blood pressure

## Discussion

Results from this analysis suggest that sociodemographic factors impact risk factors for development and progression of DR. Significant differences were noted in both HbA1c and SBP among patients of different races and between patients in low- versus high-income households. Specifically, Black patients had higher HbA1c levels and higher SBP levels compared to White patients. Despite the difference in glycemic control and blood pressure levels between the Black and White patient cohorts, there was no significant difference in the odds of having vision threatening DR among the two groups.

Disparities in rates of DR, DME, and proliferative DR between different races have been well documented in the literature, with multiple studies reporting higher likelihood of disease development and severity in Black than White patients [3, 12, 14–16]. Our data suggest that these discrepancies may be secondary to an upstream effect of race on systemic risk factors for DR rather than race on disease alone. This is further supported by previous reports of worse glycemic and blood pressure control in Black compared to White patients [12, 14]. Identifying the differences in risk factor management can therefore allow for timely, targeted intervention and risk factor reduction in this group of patients.

Of the relationships examined in our study between ethnicity and systemic risk factors, only SBP showed a significant association with ethnicity. While previous reports have reported rates of DR to be about twice as high in Hispanic compared to non-Hispanic populations, we did not observe this pattern in our results [17]. Similarly, a link between Hispanic ethnicity and presence of VTDR has been suggested by other studies after controlling for other risk factors, but our results did not convey such a relationship [12]. These conflicting results may be due to low numbers of Hispanic patients included in this study. Also, racial identity was not specified in our ethnic cohorts (e.g. Black patients were included in the non-Hispanic cohort), which could also explain the inconsistency between our results and previously published studies. As the sample population represents patients seeking care at a large Mid-Western academic retina practice, it is likely that its ethnic composition is not representative of the national population. Despite this, it is interesting to note that we still observed a statistically significant association between Hispanic ethnicity and elevated SBP, providing another possible point of intervention to prevent worsening DR in this population.

Our results also suggest that socioeconomic status not only impacts severity of risk factors for DR but severity of disease as well. Low-income patients had higher BMI levels, higher SBP, and were more likely to have VTDR compared to high-income patients. In fact, it is interesting to note that of the primary sociodemographic factors investigated in our analysis, only income and payor (which directly impacts the affordability of an individual’s health care), the two modifiable factors, were significantly associated with the presence of VT diabetic retinopathy. This implies that socioeconomic status is a primary driver of risk for DR and that many of the disparities noted among different races and ethnicities are in fact due to disparities in socioeconomic standing. Signorello and colleagues came to a similar conclusion in their study, which showed that though African American adults are 50-100% more likely compared to White adults to have diabetes, those differences in prevalence are likely due to differences in established risk factors for disease, such as socioeconomic status, which vary among the two racial groups [18]. Additional findings from a 2019 meta-analysis demonstrated an association between low neighborhood socioeconomic status and greater odds of overweight and obesity, demonstrating that health consequences cannot be attributed to individual behavior alone [19]. Low socioeconomic neighborhoods often have limited opportunities to support a healthy lifestyle, including low availability of affordable nutrient-dense foods and exercise facilities. Neighborhood factors have also previously been correlated with poorer outcomes of cardiovascular disease and mental health [19], further emphasizing the consequential impact of environment on the development and progression of chronic disease. While development of DR is likely multifactorial, with patient behavior, lifestyle, genetics, and environment all playing key roles, these findings suggest that disparities in rates and outcomes of DR may be further reduced by addressing broader social issues, such as income inequality and affordability of health insurance, and that systemic societal barriers may have a deep, longstanding impact on eye health and vision.

Money is a well-known barrier to healthcare. A systematic review of 77 studies reported that low income and financial concerns were most often reported as limitations by patients [20]. Our findings that low income is associated with higher BMI is supported by another study investigating the impact of a one- versus two-adult family structure on BMI in 7478 children [21]. Their confounder-adjusted analysis controlling for highest educational attainment and ethnicity still found income was the most significant mediating factor in BMI outcomes, reinforcing the importance of financial concerns when considering disparities in disease outcomes. Several reports have investigated potential root causes for the differences noted in disease development and progression among varying sociodemographic cohorts. Access to healthcare and financial concerns have frequently been identified as primary barriers that disproportionately impact health outcomes among certain sociodemographic groups. Routine healthcare is vital in timely identification of diabetes and diabetic retinopathy, especially given the early asymptomatic stages of disease. One three-year study found that almost 11% of patients were unaware of having diabetic retinopathy, with notable correlates including Black race, Hispanic ethnicity, and elevated blood pressure [22]. Another report showed that patients of low socioeconomic backgrounds as well as racial and ethnic minorities are less likely to receive routine eye care, most notably an annual eye exam [23]. Authors identified various structural factors responsible for this disparity, such as limited transportation options, opportunity costs associated with patient employment, and unfavorable clinical experiences. In a focus group conducted by Elam et al., clinical experiences were also cited as major contributors to healthcare disparities, namely weak patient-provider relationships, mistrust in the healthcare system to address their needs, and lack of patient-centered communication, in addition to the high copays and distant proximity to clinics [24]. Awareness of these barriers is crucial for optimizing continuity of care and health outcomes in these populations.

There are several limitations of this study. First, the data collected is from a population sample representative of those seeking care at an academic institution in southeast Michigan, allowing for selection bias and the possibility that the ethnic mix of patients in this study is not necessarily representative of other communities. Non-Hispanic patients were overrepresented in our study compared to the general US population [25]. Also, racial identity was not delineated in the non-Hispanic or Hispanic cohorts or in the unknown/other/mixed group, which warrants some caution when interpreting these data. However, of note, the racial distribution of patients, most notably Black and White patients, did closely parallel the racial demographics in the United States Census [25]. Second, the retrospective design of the study limits analysis to data already available in the electronic health record and relies on surrogate markers such as median household income based on zip code to approximate patient income. Hemoglobin A1c values were only available for 44% of subjects as many patients who received eye care at this retina practice received primary care and lab testing outside the institution. Thirdly, longitudinal analyses of the data are limited by the 40% of patients who only had one visit during our study period. Finally, data regarding duration and prior medical treatment of systemic disease were not analyzed as evaluation of risk factors for development of DR was outside the scope of this paper; however, it is important to acknowledge these as potential confounding variables. Despite this, the large sample size, a racial mix similar to that of the national population, and lack of other reports on the impacts of sociodemographic factors on risk factors for DR are important strengths of this study, which can serve as a basis for further investigation.

## Conclusion

This report demonstrates a significant association between sociodemographic factors and well-established risk factors for DR such as HbA1c, blood pressure, and BMI, and suggests that disparities in rates of DR among varying groups may be addressed by early interventions aiming to minimize those risk factors. Importantly, this study also shows that despite significant differences in risk factors for development of DR among varying racial and ethnic cohorts, the only sociodemographic factors actually associated with having vision-threatening disease were the modifiable ones—income and payor. This finding suggests that disparities noted in outcomes of disease may be further reduced by addressing broader social issues such as income inequality. The results of this short-term study underscore the importance of further longitudinal research on the association of sociodemographic factors not only with disparities in DR outcomes, but also with development and progression of DR, and the need for earlier, more targeted interventions for patients in these higher-risk groups.

## Electronic supplementary material

Below is the link to the electronic supplementary material.

Supplementary Material 1

## Data Availability

The datasets used and/or analyzed during the current study are available from the corresponding author on reasonable request.

## Abbreviations

BMI: body mass index
DME: diabetic macular edema
DR: diabetic retinopathy
HbA1c: hemoglobin A1c
SBP: systolic blood pressure
VT: vision-threatening
