# Supplementary material for: Sociodemographic variables associated with risk for diabetic retinopathy
Source: Clin Diabetes Endocrinol. 2022 Oct 24;8:7. doi: 10.1186/s40842-022-00144-z (PMC9590219; doi:10.1186/s40842-022-00144-z)
Supplement: Supplementary file 1 — Supplementary Material 1 [file 40842_2022_144_MOESM1_ESM.docx]

| **Reviewer comment** | **Changes made** | **Explanation/comments** |
| --- | --- | --- |
| Reviewer #1: The authors have satisfactorily addressed the concerns of the prior submission, which is a good step forward in the understanding of the complex factors that determine outcomes in chronic disease. The authors still imply, if not explicitly stated, that the responsibility for VT-DR lies with society rather the patient, and that income equality is achievable and a solution. This is naive and not likely to solve any problems. We are all responsible for the our actions and the consequences. | Lines 264-266 | Thank you for this comment. We have tried to emphasize that development of DR is multifactorial and complex, while also underscoring the findings of the paper that suggests the significant role that socioeconomic factors also play in creating the disparities is disease development and progression often noted among differing racial and ethnic groups. |
| An interesting read, thank you. Despite study design limitations and unclear handling of missing data, the conclusion that only income/payor were associated with VTDR are interesting. This needs to be emphasised as a key contribution of this study to the literature, given the associations between low income/ethnic background and poor health outcomes are already well-known.  Further expansion of the significance of payor is needed (see specific comments below**). Please also change HgbA1c to HbA1c as the commonly-accepted abbreviation. Similarly sBP should be SBP and VT-DR simply VTDR.** | Changed as requested |  |
| Line 21: The hypothesis needs to be more specific - which sociodemographic factors did you hypothesize affecting systemic DR risk factors? These are listed under the methods but ideally should be listed here to make the hypothesis clear to the reader. | Changed as requested. Lines 22-23 |  |
| Line 121: Please clarify the source(s) of referral for patients presenting to the retina clinic. Who identified them as having DR requiring a clinic review? E.g. population-based screening program, family practitioner, internal medicine, etc? Do you have any data on the time from date of referral to review in clinic? This is important to understand whether some patient groups had worse disease due to a delayed presentation to secondary/specialist care. | N/A | Thank you for this comment. Regrettably, the CDRP does not have data available regarding sources or timing of patient referral this this time.  While delayed presentation is undeniably an important factor in disease severity, our aim was to examine the impact of sociodemographic factors on systemic risk factors of DR and on presence of VTDR. An analysis that associates factors with development of VTDR falls outside the scope of our study.  Diagnosis of VTDR was made using billing codes associated with patient encounters, as stated in lines 137-141 |
| Line 130: Did the non-Hispanic cohort include Black patients? This may explain why you did not observe a difference in DR rates. For a more meaningful comparison, please compare Hispanic White vs non-Hispanic Whites. If not, then state this as a limitation of the study in the relevant discussion section (lines 231-237). | Clarified in the text, lines 236-238 and 298-299. | Thank you for this comment. This brings up a valid point that has been clarified in the discussion and limitation sections of the text. |
| Line 148: Linear mixed models are appropriate for this dataset. Please confirm if residuals were plotted and any patterns observed, to verify homoscedasticity, outliers, normality, etc. | Lines 150-151 | Thank you for this comment. Fit diagnostics were plotted and residuals demonstrated no pattern. This has been confirmed/clarified in the text. |
| Line 155: What is the ethnicity/racial mix of the general population? Although racial distribution is mentioned in the discussion (lines 292-294), these denominators need to be added to the paper in order to understand whether certain groups are over-represented in the DR cohort. | Clarified in the text, lines 297-299. | Thank you for this comment. Non-Hispanic patients were overrepresented in our study compared to the general US population. This has been added in lines 297-299. |
| Line 164: Do you have any data on medication and durations of disease? E.g. % taking antihypertensive medication, duration of diabetes (<1 year, 1-5 years, >5 years). If not then please state this, as these are confounding factors when interpreting the data. For example, White patients might be more compliant with treatment than Black patients, hence why their HbA1c/SBP were lower. This should also be mentioned in the relevant discussion section (lines 227-230). | Clarified in the text, lines 309-310. | Thank you for this comment. While systemic disease duration and control are important confounding factors to consider in the development of DR, our hypothesis was built around the presence of systemic disease and VTDR alone and thus this data was not collected. This has been clarified. |
| Do you have the exact breakdown of unknown/other/mixed under race and unknown/refused under ethnicity? Some caution is needed in interpreting these data, as there is likely to be an underlying bias (e.g. some ethnic minorities may be less willing to identify themselves, leading to a selection bias). This should be toned down in the results and removed from the discussion (line 219) for the reasons mentioned. | Removed from the text, line 219. Clarified in the text, line 294-295. | Thank you for this comment. Though we are able to present a breakdown of race and ethnicity under the “unknown/other” category, we did not feel that this information would significantly impact our results/findings, in part due to the concern for underlying bias as referenced by the reviewer. This has been clarified in the text.  While we believe it important to present our findings in the results, we have removed them from the discussion section. A note in the Limitations has also been made. |
| Line 245: What is the significance of the association between payor and VTDR? This needs to be expanded in the discussion, particularly for the non-US reader. | Line 249 | We state that income and payor imply socioeconomic impact in lines 245-250. We have added a short clarification on the implication of “payor” on line 249 for non-US readers. Additional discussion of impacts of income in lines 268-291 |
| Line 263: 'Longstanding' should not be hyphenated. | Changed as requested |  |
| Line 283: Some excellent points discussed here. Physician mistrust, health misinformation and distance from clinic are well-known associations with poorer health outcomes. What about insurance coverage? This is not mentioned in the paper. Whilst data might be difficult to obtain for obvious reasons, it should be mentioned that the true scale of health inequalities, particularly among low-income groups, is likely to be underestimated. | N/A | Thank you for this comment. Insurance has been discussed in the text as mentioned above. We also extensively discuss health inequalities among low-income patients in lines 270-293. |
| Line 314: Please briefly describe what a future study might look like to add to our current understanding. | Clarified in the text, line 326-327. | Our short-term study has yielded important initial findings, and we anticipate further important discoveries with longitudinal studies in the future. |
